# New Empirical Equation for the Atomic Form Factor Function in the Momentum Transfer Range, q = 0–50 Å^−1^ for the Elements in the Range 1≤ Z ≤30

**DOI:** 10.1371/journal.pone.0069608

**Published:** 2013-08-01

**Authors:** Wazir Muhammad, Sang Hoon Lee

**Affiliations:** 1 Department of Medical Physics, Institute of Nuclear Medicine Oncology and Radiotherapy (INOR), Abbottabad, Pakistan; 2 Department of Physics, Kyungpook National University, Daegu, Republic of Korea; 3 School of Energy Engineering, Kyungpook National University, Daegu, Republic of Korea; University of Leeds, United Kingdom

## Abstract

The importance of Atomic Form Factors (f) is well-known to the scientific community. Tabulated values for f are mostly used in calculating cross-sections and Monte Carlo sampling for the coherent scattering of photons. The uses of these values are subjected to different approximations and interpolation techniques because the available data points for f in the literature for specified momentum-transfer-grids are very limited. In order to make it easier to accurately use the tabulated data, a mathematical expression for f functions would be a great achievement. Therefore, the current study was designed to suggest an empirical expression for the f functions. In the results, an empirical equation for Hubbell's tabulated data for f is created in the momentum transfer range, q = 0–50 Å^−1^ for the elements in the range 1≤ Z ≤30. The number of applied parameters was seven. The fitting to f showed that the maximum deviation was within 3%, 4% and 5% for the element having, Z = 1–11, Z = 12–22 and Z = 23–30, respectively, while the average deviations were within 0.3–2.25% for all elements (i.e., Z = 1–30). The values generated by the analytical equation were used in the Monte Carlo code instead of Hubbell’s tabulated values. The statistical noise in the Probability Distribution Functions of coherently scattered photons was efficiently removed. Furthermore, it also reduced the dependence on different interpolation techniques and approximations, and on the use of large tabulated data for f with the specified elements.

## Introduction

The Rayleigh (elastic) scattering of photons by a bound atomic electron is one of the major modes of interaction of photons with matter particularly for low energy x-rays and soft γ-rays. It has been used as a valuable investigative tool for quite a long time in various fields (i.e., structural chemistry/biology,?shielding and medical diagnostics, etc.) [1]–[4].

Atomic form factors (f) are important factors in the Theory of Rayleigh scattering for photons [1]. Precise values of f for any element have long been required because of their importance and applications in fundamental science as well as in the various applied fields such as material study, health physics, biology, medicine, etc. Owing to its importance, successful efforts have been made to calculate their extensive and complete tabulations. In the last quarter of the 20th century, these tabulations have been published for the neutral atoms in the Periodic Table having Z = 1 ∼ 100 in the various energy ranges [5]–[7]. Present, the state-of-the-art method (i.e., S-matrix; SM) has been developed to estimate the most precise f. However, these techniques require a large computer and extensive CPU time [8]. The f are traditionally tabulated as a function of momentum transfer, 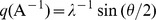 (i.e., λ (Å) is the incident photon wavelength). These tables are extremely useful and widely used.

In the literature, one can find extensive tabulations for f and theoretical calculations for the Rayleigh scattering of photons. Some of these are publicly available and some are distributed for specific users in the form of data libraries (i.e. EPDL97, RTAB database etc.). One such database is known as EPDL, the Evaluated Photon Data Library 1997 version (EPDL97) [9]. EPDL97 is part of the ENDF/B-VII.1 [10]. It contains evaluated nuclear data file, total cross-sections, f and Anomalous Scattering Factors (ASFs). By using the numerical integration method, the total Rayleigh cross-sections are derived from the Thomson scattering, f and ASFs. It uses different estimations for such calculations but the details of these estimations cannot be found in the literature. EPDL97 is extensively used in MC simulation packages. A set of photon Rayleigh scattering cross-section tabulations, based on f and ASFs is also included in RTAB [11]. The differential cross-sections tabulated in RTAB are based on Non-relativistic Form Factors (NFFs) by Hubbell et al., Relativistic Form Factors (RFFs), Modified Form Factors (MFFs), Numerical S-matrix calculations by Kissel and Pratt, and the MFF+ASF scheme.

Monte Carlo (MC) simulation is the most adequate tool to trace the interaction between photons and atoms. Numerous MC based computer codes are currently available for photon transport having coherent scattering as its integral part (i.e., GEANT4, EGSnrc, MCNP, PENELOPE, etc.) [12]–[15]. To the best of the authors’ knowledge, the most widely used tabulated data for f in many of these MC packages to simulate coherent scattering of photons are from Hubbel and Øverbø {i.e., 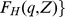 [5]. The 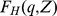 (i.e. for momentum transfer q = 0–10^9^ Å^−1^ for all elements Z = 1–100) have been constructed from available theoretical partial-range tabulations and in some cases, from computations using formulas given in the literature [5]. In addition, different theoretical equations and atomic models have been used in the formation of these tabulated data. There are 100 data points available in the above q-grid.

Use of tabulated values for f in practical calculations or in applications for MC simulation packages may lead to some difficulties because a great amount of data has to be used [16]. Solutions to these difficulties have been attempted using different interpolation techniques. Furthermore, in MC simulation packages, use of complete tables for f which cover the entire energy range is almost impossible or can be cumbersome some times, especially if these are included in a large production code where the speed of execution is of primary importance [17]. A popular method in the literature so far is the Linear Interpolation Technique (LIT) which evaluates and uses tabulated data in MC simulation packages [13], [15]. However, statistical noise is produced when LIT is used in MC sampling techniques for coherently scattered photons [17].

Theoretically, the most consistent treatment of Rayleigh scattering is to use RFFs with ASFs to define the coherent scattering cross-section [9], [18]. It is worthwhile to produce some empirical expressions to approximate these tabulated values of 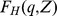 to overcome these difficulties and shortcomings for applications in different fields. Many studies, well-summarized by Imre Szaloki [16], can be found in the literature that deal with these empirical expressions and are summarized as follows [16], [19]–[28]:



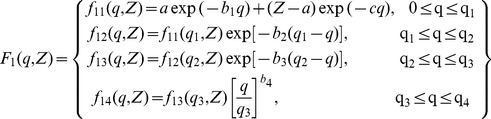
(1)


For 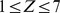 and similarly, for 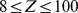 [16]:
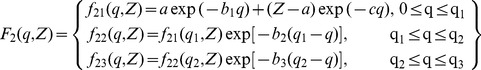
(2)



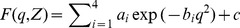
(3) [19]–[22], [24]–[28]


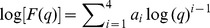
(4) [23]

A combination of analytical functions shown in equations (1) and (2) has been fitted by Imre Szaloki [16] to the data for 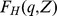 calculated by Hubbel and Øverbø [5]. A total of 10 and 8 fitting parameters have been used in equations (1) and (2), respectively. The maximum deviations are within 10% except for Z = 3, 6, 7, 8 and 9. The momentum transfer range is q = 15 Å^−1^ for Z = 1, 34–48, q = 10 Å^−1^ for Z = 16–33, 80–100, q = 8 Å^−1^ for Z = 2–7, 60–79, and q = 7 for Z = 8–15, 49–59 while for equations (3) and (4), q = 0–2 Å^−1^. The feasibility of equation (1) and (2) is uncertain because of both the covered momentum range and fitting accuracy. The most accurate fit was achieved for Equation (3) by applying 9 fitting parameters [20], [28] but the range of q was very small (i.e., q = 0–2 Å^−1^). On the other hand, a rational function with only 4 fitting parameters was applied in Ref. 16 for q = 0–8 Å^−1^. However, it had very high deviations (20–50%) between the tabulated and calculated data in some cases [16].

The importance of f for all elements listed in the periodic table and for a wide energy range is well-known to the scientific community. Currently, tabulated values for f are mostly used in calculating cross-sections and MC sampling for coherent scattering of photons. In the literature, the available data points for f in the above specified q-grid are very limited (i.e. 100 points). To cover a continuous range of q, the usual various interpolation techniques have been used, but these techniques have their own limitations [17]. In order to make it easier to accurately use widely used tabulated data for 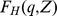 [5], a mathematical expression for f functions would be great achievement. Previous efforts in this regard have highlighted the importance of this matter. In addition, mathematical models of coherent scattering have also been used to construct a single equation for form factor and scattering functions [16]. On the other hand, from the literature, previous approximations have some limitations in both the q-grid and fitting accuracy. These factors motivated the current study to suggest a new mathematical expression for the 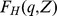 function fitting.

## Modeling of the Empirical Function

In order to model an empirical 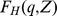 function, some basic considerations were taken. These include the following: (1) a single analytical function should cover the maximum value of q; (2) the function should have the smallest possible number of parameters and (3) the fitting accuracy should be as high as possible (i.e. the maximum % deviation limit; 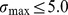). The existing analytical function for the hydrogen atom was taken as a base line to construct the general equation for elements having Z = 1 to 30. The analytical equation for the hydrogen atom is as follow [5]:
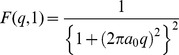
(5)


The 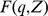 function was constructed from the available analytical equation for Hydrogen as stated above, depending on the value of the atomic number Z as follows:
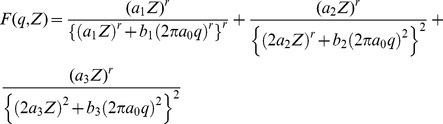
(6)where; 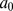 = 0.52978 Å represents the Bohr radius while r, a_1_, a_2_, a_3_, b_1_, b_2_, and b_3_ are the fitting parameters. The function is accurately fitted in the q-grid from 0 to 50 Å^−1^. Non-linear curve fitting tools in Origin 8 were used to calculate these parameters.

The % deviation from 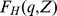 was calculated using the following relation [16]:
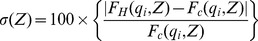
(7)


Here, q_i_ is the momentum transfer grid values given in Hubbel and Øverbø [5], 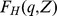 represent Hubbell's data depending on Z and 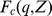 are the calculated values of the fitted analytical functions used in the present work. The maximum, 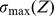, mean 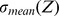 and standard deviation, 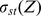 of the % deviation between the calculated and tabulated data for each element were calculated accordingly.

## Application in MC Code

For the practical application of the designed empirical function, the code by Wazir Muhammad and Sang Hoon Lee [17], [18] for MC sampling of coherent scattering of photons with modification for the analytical function was used. The sampling techniques remained the same but instead of using the tabulated values for 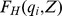, the integrated values of 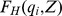 squared (i.e.,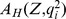 over 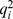) and the corresponding values of the q-grid (i.e. q = 0 ∼ 16 Å^−1^), using the modeled function given in Equation (5), were added to the code. By executing the code for a particular element, new values of 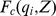 with the help of the modeled function were generated. Furthermore, the integrated values of 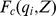 squared against *q^2^* were calculated by another function included in the calculation of 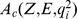. These steps are done at each code’s run for the MC simulation of coherent scattering of photons.

To study the impact of the newly modeled analytical function on the MC sampling of coherently scattered photons, 1^st^ PDF were constructed through 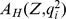 for 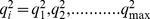 and hen through 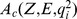 for 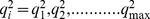 using the above mentioned MC techniques. The MC study was performed for Hydrogen (H, Z = 1), Sodium (Na, Z = 11), Silicon (Si, Z = 14) and Manganese (Mn, Z = 25).

## Results and Discussion

**Figure 1:**
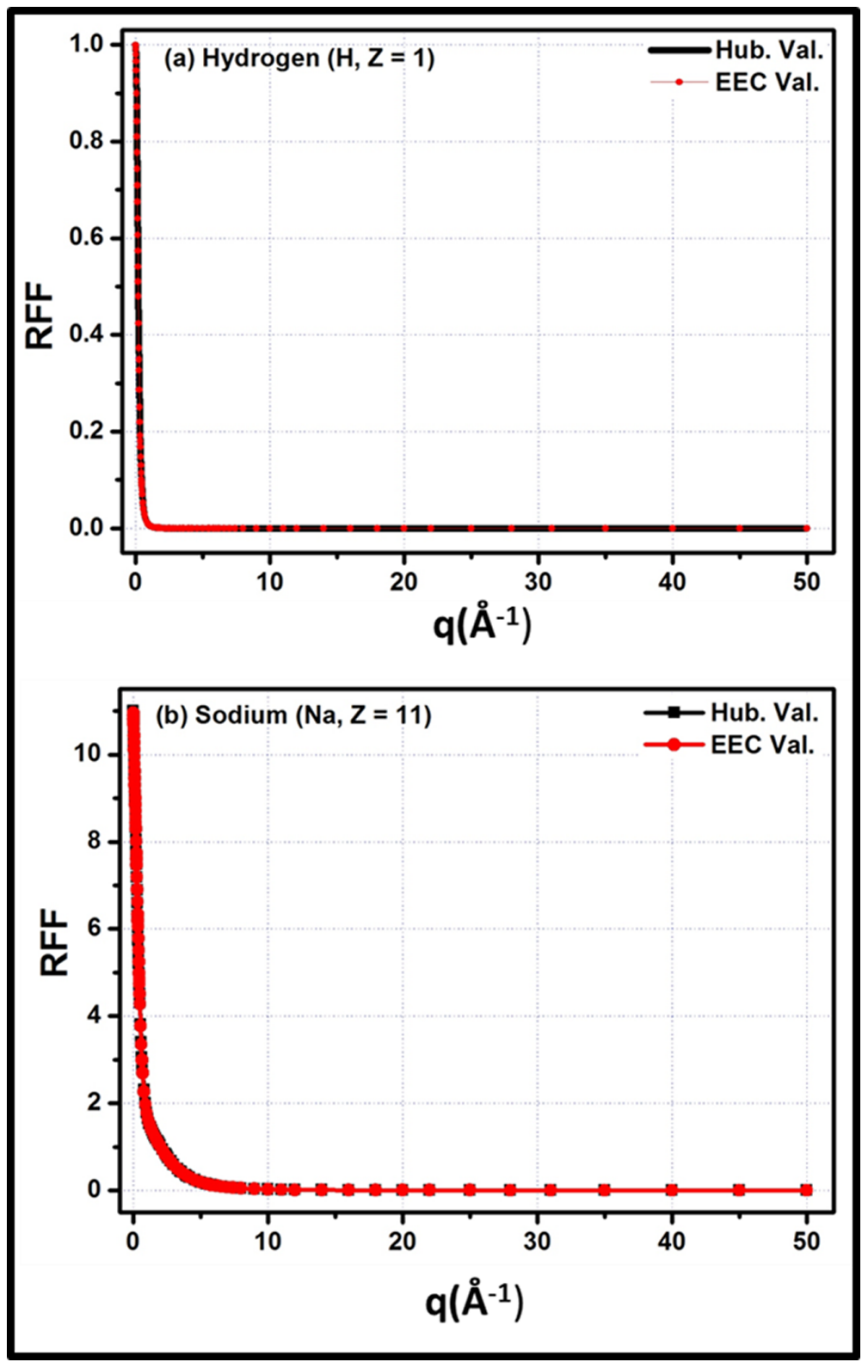
Fitting of the 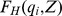 function in the momentum transfer range, q = 0–50 Å^−1^ for (a) Hydrogen (H, Z = 1) (b) Sodium (Na, Z = 11). Here, Hub. Val. Indicates 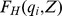 and EEC val. indicates Empirical Equation Calculated values.

**Figure 2:**
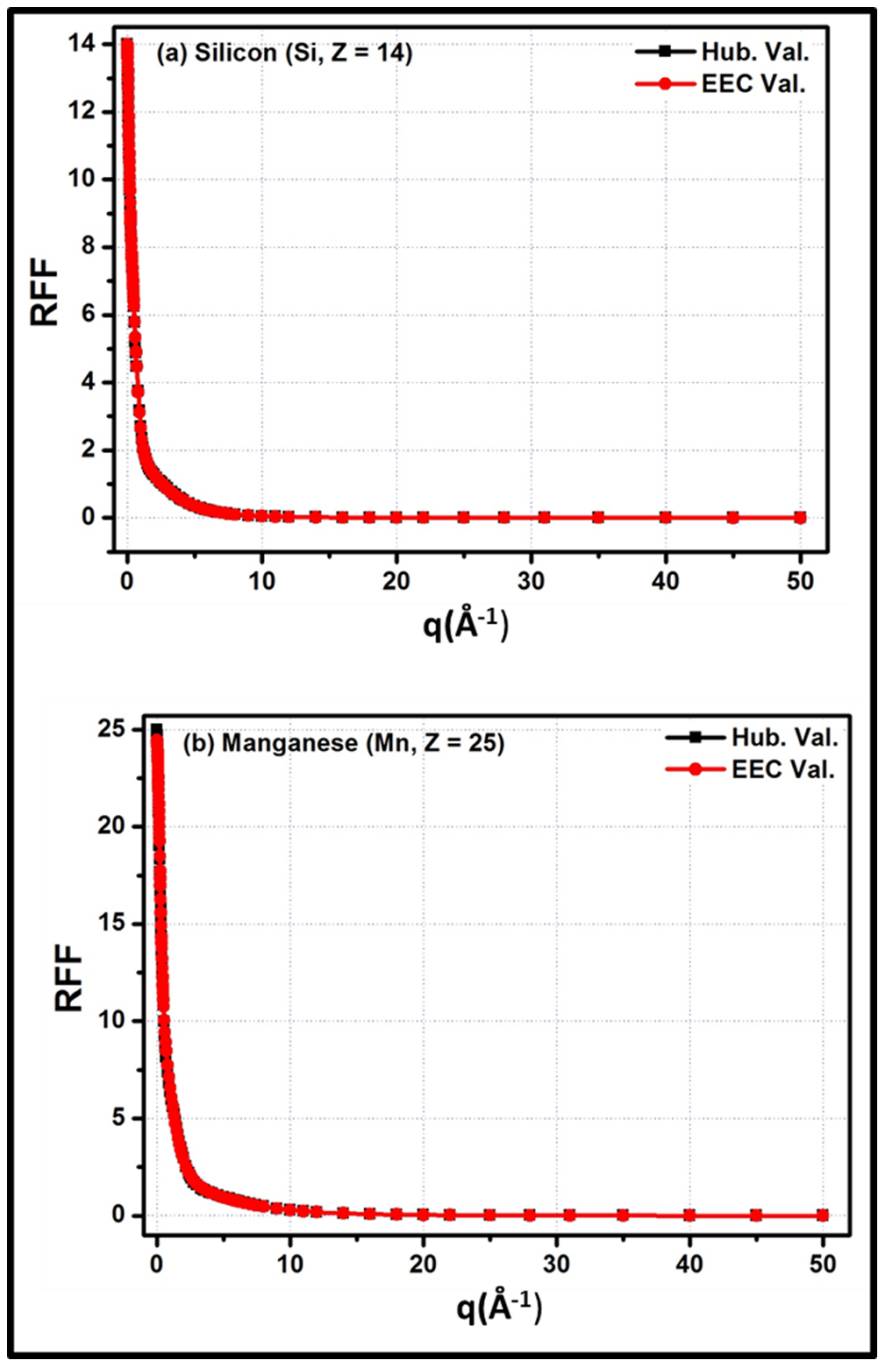
Fitting of the 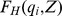 function in the momentum transfer range, q = 0–50 Å^−1^ for (a) Silicon (Si, Z = 14) (b) Manganese (Mn, Z = 25). Here, Hub. Val. Indicates 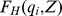 and EEC val. indicates Empirical Equation Calculated values.

The calculated fitting parameters (i.e. r, a1, b1, a2, b2, a3 and b3) along with 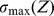, 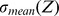 and 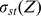 corresponding to each element, are summarized in Table 1. Equation 6 is reduced to equation 5 by taking r = 2, a_1_ = b_1_ = b_2_ = b_3_ = 1 and a_2_ = a_3_ = 0 for all values of q except q = 0 at which the 2^nd^ and 3^rd^ term become undefined. To avoid this trap, a_2_ = a_3_ = 1E5 is taken. The results shows that 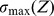 from 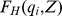 are within 3%, 4% and 5% for elements having Z = 1–11, Z = 12–22 and Z = 23–30, respectively while the 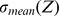 and 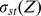 are within 0.3–2.25% and 0.15–1.35% respectively for the listed elements (i.e., Z = 1–30). Figures 1 and 2 are examples of the fitting of the empirical equation with 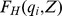 for Hydrogen (H, Z = 1), Sodium (Na, Z = 11), Silicon (Si, Z = 14) and Manganese (Mn, Z = 25) by utilizing the parameters listed in table 1 for these elements. The figures show that the empirical equation is well fitted to 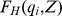 functions. To the best of the authors’ knowledge, this is the 1^st^ empirical equation which is valid for such a large q-grid with a higher accuracy compared to previous research. According to the designed % deviation limit (i.e. 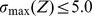) of this study, the current empirical equation has some limitations in terms of Z (i.e. 1≤ Z ≤30). Beyond this limitation, the fitting is not worse; however, 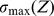 crosses the design limit of the study. Furthermore, f are being used to calculate the cross-sections for coherent scattering. Normally, different approximations with interpolation techniques and large tabulated data are used to calculate the cross-sections. The function will be very useful in calculating the cross-section for the Rayleigh scattering of photons and reduce the dependence on different approximations and interpolation techniques while calculating these cross-sections. Apart from these advantages, it will also reduce the dependence on large tabulated data for f.

**Table 1:** Parameters of the function F_H_(q, Z) corresponding to Equation (6).

Z	r	a_1_	b_1_	a_2_	b_2_	a_3_	b_3_	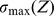	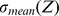	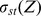
1	2.0	1	1	1E5	1	1E5	1	0.49407	0.30869	0.16277
2	1.994	1.07342	0.64272	0.12568	0.0845	0.14111	0.17253	2.95168	0.77506	0.97183
3	2.934	0.38714	0.09257	0.07786	0.554	0.01882	0.00128	2.80411	0.82839	0.47785
4	2.998	0.28152	0.01825	0.04686	0.1465	0.01342	5.8E-4	2.95438	1.19439	0.74471
5	2.9952	0.2303	0.00808	0.03316	0.06074	0.01083	3.69E-4	2.83182	1.15413	0.76625
6	2.87	0.18732	0.00503	0.02471	0.03378	0.01421	5.79E-4	2.91821	1.27202	0.93683
7	3.0808	0.13286	0.2496	0.02926	0.00127	0.00181	7.6E-4	2.57067	1.26022	0.67697
8	2.5968	0.0939	0.13992	0.02472	0.00135	0.00813	0.01953	2.52609	0.96599	0.93648
9	2.676	0.08332	0.09631	0.02226	0.00101	0.00534	0.00698	2.76167	1.30524	0.83583
10	2.6768	0.07474	0.0664	0.02016	8.15E-4	0.004	0.00361	2.97721	1.32855	0.94562
11	2.4892	0.0539	0.0382	0.01797	7.56E-4	0.00855	0.11457	2.9295	1.10247	0.95764
12	2.573	0.05214	0.02571	0.01662	5.95E-4	0.00493	0.03657	3.02582	1.03358	1.00879
13	2.5948	0.04874	0.01839	0.01539	4.96E-4	0.00361	0.02043	3.19991	0.96117	1.05342
14	2.7396	0.04866	0.01224	0.01441	3.82E-4	0.00192	0.00521	3.36012	0.94039	1.03047
15	2.8128	0.04704	0.00832	0.01354	3.14E-4	0.00121	0.00187	3.62753	0.88091	1.05631
16	2.9188	0.0459	0.00561	0.01276	2.54E-4	6.6E-4	5.16E-4	3.6664	0.84291	1.03753
17	3.0348	0.04484	0.00368	0.0121	2.06E-4	3.104E-4	1.059E-4	3.85843	0.84603	1.05476
18	3.1704	0.0439	0.00232	0.01151	1.66E-4	1.073E-4	1.188E-5	3.97441	0.86797	1.0771
19	3.1948	0.0416	0.00176	0.01105	1.48E-4	7.923E-5	6.49E-6	3.85914	1.55547	1.10554
20	3.1412	0.03902	0.00155	0.01046	1.38E-4	1.059E-4	1.21E-5	3.9819	1.79997	0.95174
21	2.5908	0.03036	0.00331	0.00964	1.83E-4	0.00112	0.0016	3.95707	1.98217	1.083
22	2.5368	0.02814	0.00307	0.00923	1.73E-4	0.00116	0.00172	3.98871	2.09126	1.16429
23	2.7128	0.02944	0.00212	0.00877	1.36E-4	6.294E-4	4.761E-4	4.44428	1.99762	1.22413
24	2.7208	0.02866	0.00167	0.00854	1.26E-4	5.428E-4	3.244E-4	4.23959	1.68691	1.0518
25	2.536	0.02488	0.00207	0.00813	1.32E-4	8.906E-4	9.251E-4	4.80743	2.1238	1.15358
26	2.58	0.02458	0.00171	0.00785	1.18E-4	7.26E-4	5.92E-4	4.83153	2.05015	1.15511
27	2.632	0.02438	0.00137	0.00763	1.05E-4	5.73E-4	3.55E-4	4.81653	2.05999	1.19439
28	2.648	0.02378	0.00123	0.00727	9.46E-5	5.05E-4	2.69E-4	4.85159	1.98741	1.23426
29	2.648	0.02328	0.00102	0.00712	8.91E-5	4.61E-4	2.12E-4	4.23183	1.73644	1.1373
30	2.78	0.02398	7.35E-4	0.00677	7.09E-5	2.304E-4	5.28E-5	4.96515	2.22336	1.32416

**Figure 3:**
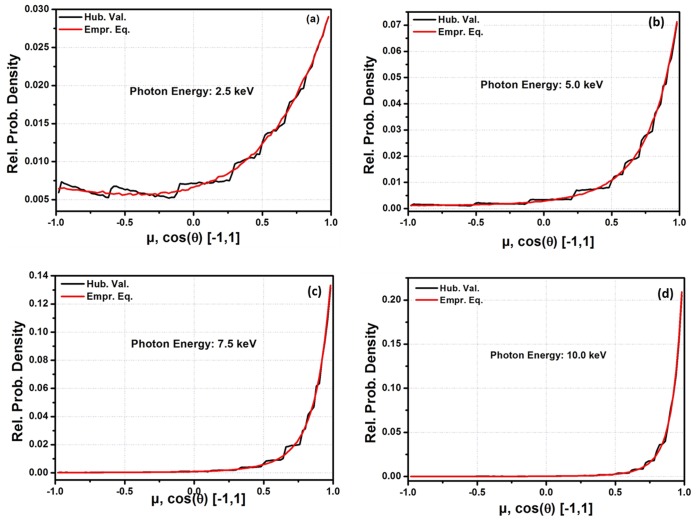
Angle distribution of coherently scattered photons for a source of 1 million photons by using 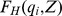 for ‘*q*’ from 0 to 16.0 Å^−1^ and Empirical equation data for Hydrogen (H, Z = 1) as a scatter element. (a) energy = 0.0025 MeV, (b) energy = 0.005 MeV, (c) energy = 0.0075 MeV, (d) energy = 0.01 MeV.

**Figure 4:**
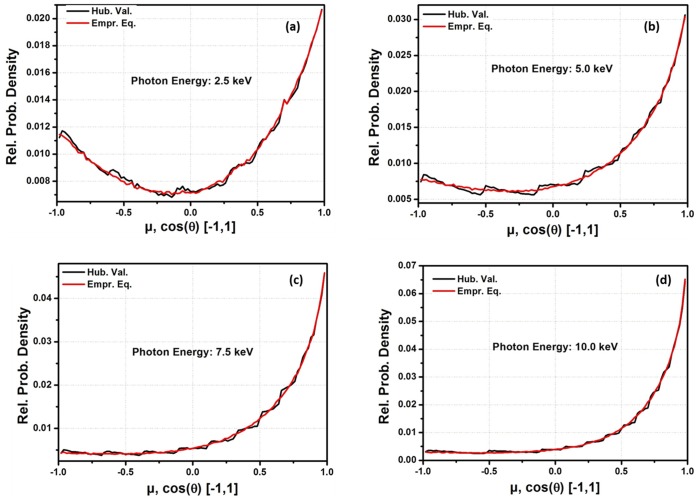
Angle distribution of coherently scattered photons for a source of 1 million photons by using 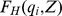 for ‘*q*’ from 0 to 16.0 Å^−1^ and Empirical equation data for Sodium (Na, Z = 11) as a scatter element. (a) energy = 0.0025 MeV, (b) energy = 0.005 MeV, (c) energy = 0.0075 MeV, (d) energy = 0.01 MeV.

**Figure 5:**
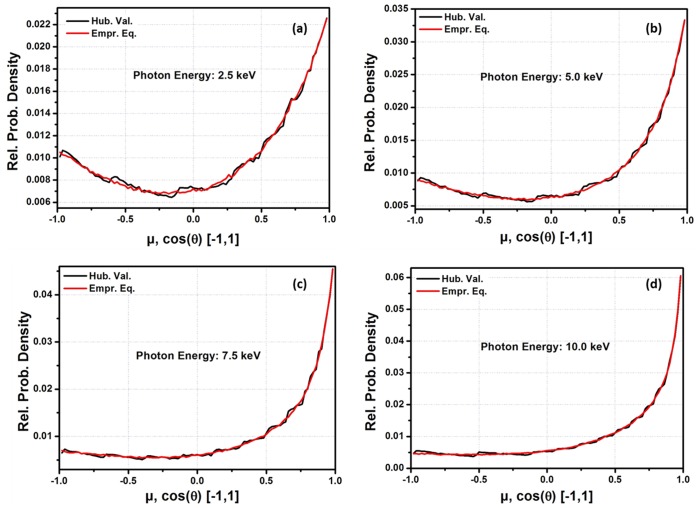
Angle distribution of coherently scattered photons for a source of 1 million photons by using 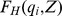 for ‘*q*’ from 0 to 16.0 Å^−1^ and Empirical equation data for Silicon (Si, Z = 14) as a scatter element. (a) energy = 0.0025 MeV, (b) energy = 0.005 MeV, (c) energy = 0.0075 MeV, (d) energy = 0.01 MeV.

**Figure 6:**
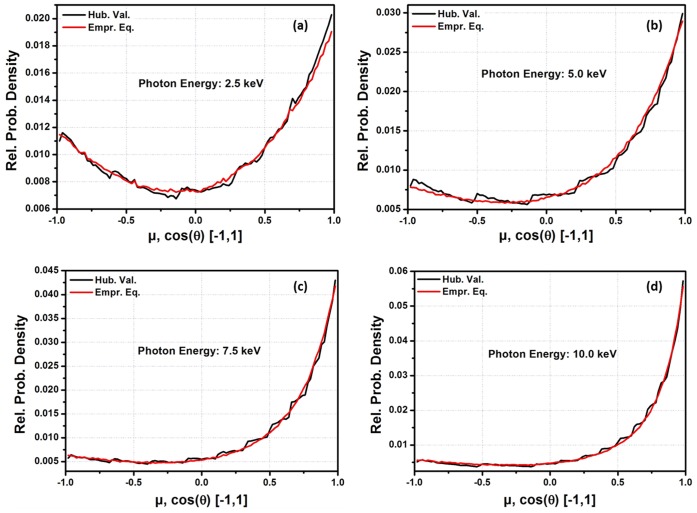
Angle distribution of coherently scattered photons for a source of 1 million photons by using 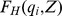 for ‘*q*’ from 0 to 16.0 Å^−1^ and Empirical equation data for Manganese (Mn, Z = 25) as a scatter element. (a) energy = 0.0025 MeV, (b) energy = 0.005 MeV, (c) energy = 0.0075 MeV, (d) energy = 0.01 MeV.

One of the major areas in the application of f is in MC codes for coherently scattered photons. For practical applications, the empirical equation was applied to an MC code designed to sample the angular distribution of coherently scattered photons. Figures 3a–d, 4a–d, 5a–d and 6a–d show the probability densities for coherently scattered photons, with 2.5 keV, 5.0 keV, 7.5 keV and 10.0 keV energies for Hydrogen (H, Z = 1), Sodium (Na, Z = 11), Silicon (Si, Z = 14) and Manganese (Mn, Z = 25) as scattering elements based on 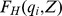 for ‘q’ from 0 to 16.0 Å^−1^ and on the currently developed empirical equation. The results show that the density of statistical noise is significantly reduced with the application of the empirical equation for the MC sampling of coherently scattered photons. The results still had some statistical noise in some cases because the empirical equation is only used to generate the f data with the smallest possible interval lengths during each code run. The generated data is further used by the code for the simulation. It means that the code is still using LIT. It is possible that statistical noise can be efficiently removed if the empirical equation is used in the sampling techniques rather than generating the f data. This is one of the advantages of using the empirical equation that it is very helpful in minimizing the statistical noise in the PDFs for the angular distribution of the scattered photons. Furthermore, large tabulated data are needed for each element in these MC packages. For example, the current MC code needs a total of 85 data points for the q-grid (from 0 to 16 Å^−1^). To be more specific, a total of 190 entries are required for each element in the f data base. However, the empirical equation reduced these 190 entries to just 7 entries (i.e. fitting parameters for each element) and the results were also improved significantly.

### Conclusion

The newly formed empirical equation was fitted to Hubbell's f tabulated data in the momentum transfer range, q = 0–50 Å^−1^ for elements in the range 1≤ Z ≤30. The number of applied parameters was seven. In conclusion, the empirical equation is well fitted to 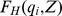 functions in comparison to its validity range of the q-grid and Z and number of applied parameters. The empirical equation is very helpful in minimizing the statistical noise of the PDFs for the angular distribution when applying it in the MC code for coherently scattered photons. Furthermore, it can be very helpful in many other applications for example, calculation the total cross-section for the coherent scattering of photons etc. In addition, it will also reduce the dependence on different interpolation techniques and approximations and on the use of large tabulated data for f.
